# Activins and their related proteins in colon carcinogenesis: insights from early and advanced azoxymethane rat models of colon cancer

**DOI:** 10.1186/s12885-016-2914-9

**Published:** 2016-11-11

**Authors:** Bassem Refaat, Adel Galal El-Shemi, Amr Mohamed Mohamed, Osama Adnan Kensara, Jawwad Ahmad, Shakir Idris

**Affiliations:** 1 Laboratory Medicine Department, Faculty of Applied Medical Sciences, Umm Al-Qura University, Al-Abdeyah, PO Box 7607, Makkah, Kingdom of Saudi Arabia; 2 Department of Pharmacology, Faculty of Medicine, Assiut University, Assiut, Egypt; 3 Clinical Laboratory Diagnosis, Department of Animal Medicine, Faculty of Veterinary Medicine, Assiut University, 71526 Assiut, Egypt; 4 Clinical Nutrition Department, Faculty of Applied Medical Sciences, Umm Al-Qura University, Al-Abdeyah, PO Box 7607, Makkah, Kingdom of Saudi Arabia

**Keywords:** Colon cancer, Activin-A, Activin-AB, Smads, Follistatin and carcinogenesis

## Abstract

**Background:**

Activin-A may exert pro- or anti-tumorigenic activities depending on cellular context. However, little is known about its role, or the other mature activin proteins, in colorectal carcinoma (CRC). This study measured the expression of activin βA- & βB-subunits, activin type IIA & IIB receptors, smads 2/3/4/6/7 and follistatin in CRC induced by azoxymethane (AOM) in rats. The results were compared with controls and disseminated according to the characteristics of histopathological lesions.

**Methods:**

Eighty male Wistar rats were allocated into 20 controls and the remaining were equally divided between short ‘S-AOM’ (15 weeks) and long ‘L-AOM’ (35 weeks) groups following injecting AOM for 2 weeks. Subsequent to gross and histopathological examinations and digital image analysis, the expression of all molecules was measured by immunohistochemistry and quantitative RT-PCR. Activin-A, activin-B, activin-AB and follistatin were measured by ELISA in serum and colon tissue homogenates.

**Results:**

Colonic pre-neoplastic and cancerous lesions were identified in both AOM groups and their numbers and sizes were significantly (*P* < 0.05) greater in the L-AOM group. All the molecules were expressed in normal colonic epithelial cells. There was a significantly (*P* < 0.05) greater expression of βA-subunit, IIB receptor and follistatin in both pre-neoplastic and cancerous tissues. Oppositely, a significant (*P* < 0.05) decrease in the remaining molecules was detected in both AOM groups. Metastatic lesions were only observed within the L-AOM group and were associated with the most significant alterations of all molecules. Significantly higher concentrations of activin-A and follistatin and lower activin-AB were also detected in both groups of AOM. Tissue and serum concentrations of activin-A and follistatin correlated positively, while tissue activin-AB inversely, and significantly with the numbers and sizes of colonic lesions.

**Conclusions:**

Normal rat colon epithelial cells are capable of synthesising, controlling as well as responding to activins in a paracrine/autocrine manner. Colonic activin systems are pathologically altered during tumorigenesis and appear to be time and lesion-dependent. Activins could also be potential sensitive markers and/or molecular targets for the diagnosis and/or treatment of CRC. Further studies are required to illustrate the clinical value of activins and their related proteins in colon cancer.

**Electronic supplementary material:**

The online version of this article (doi:10.1186/s12885-016-2914-9) contains supplementary material, which is available to authorized users.

## Background

Colorectal cancer (CRC) is a common malignancy associated with high mortality rates [1]. Several treatment modalities for CRC are available and include surgery, chemotherapy and/or radiotherapy [2, 3]. Nevertheless, the success and five year survival rates following the use of the different therapeutic approaches are mainly dependent on early diagnosis/intervention, since the majority of treatment regimens are associated with limited efficacy and relatively low survival rates during advanced stages of CRC [2, 3]. Additionally, resistance against several recently introduced chemotherapeutic agents in combination with 5-Fluorouracil has been reported by many clinical trials, rendering chemotherapy ineffective in a substantial number of patients [4, 5]. Therefore, a better understating of the biology of CRC and its underlying pathophysiological pathways is essential for the development of alternative/complementary effective therapeutic strategies [6, 7].

Several molecular pathways are pathologically skewed during colon tumorigenesis [8]. Among these pathways, the members of transforming growth factor (TGF)-β family have recently been suggested as potential stage-dependent targets for the treatment of CRC and/or prevention of resistance associated with chemotherapy [9]. Activins belong to the TGF-β family and the mature activins consist of hetero- or homodimers of 2 β-subunits (βA and βB) resulting in three distinct proteins named activin-A (βA-βA), activin-B (βB-βB) and activin-AB (βA-βB) [10]. The canonical pathway for activins, following the activation of their type II receptors (ACTRIIA & ACTRIIB), shares the same intracellular mediators with TGF-β and both are dependent on smad2, 3 and 4 [11]. Several extra- and intracellular mechanisms for the control of activins bioactivities have been described. Extracellular neutralising molecules include the well-established activin binding protein, follistatin, which binds the three mature isoforms of activin with similar affinity and prevents their interactions with type II receptors [12]. Physiological intracellular inhibitors of activins and TGF-β signals are known as inhibitory smads (smad6 & 7) and both inhibit the phosphorylation of receptor smads (smad2 & 3), prevent their interactions with smad4 as well as induce the degradation of activated activin and TGF-β type I receptors [11].

In vitro studies suggested anti-tumour activities for activin-A on several colon cancer cell lines through smad2/3/4 pathway [13, 14]. The results from human studies have further shown that malignant enterocytes develop resistance to activin mainly by inducing mutations in the activin type IIA receptor or the smad4-dependent pathway [15, 16]. Restoration of the receptor in vitro resulted in smad4-dependent growth inhibitory effects and cell cycle arrest of cancerous enterocytes but also induced their migration [17, 18]. Other studies in human have also outlined that the serum concentrations of activin-A correlate positively with tumour size, progression, invasiveness and inversely with survival rates [19–21].

At the present time, none of the available in vitro and human studies measured the role(s) of the other mature activin isoforms and/or follistatin in colonic malignancies. Additionally, there is no data in the literature on the expression of activins and their related molecules in experimental animal models of CRC. Azoxymethane (AOM)-induced CRC in rodents is a well-established and commonly used model for the study of the molecular biology, prevention and treatment of CRC. This model imitates highly similar histopathological features and shares similar molecular pathways to the sporadic phenotype of CRC in human and, adenocarcinoma usually develops after 14 weeks of AOM injection in rodents [8, 22].

The present study therefore measured the expression of activin β-subunits, type II receptors, smads 2/3, smad4 and smads 6/7 at the gene and protein levels in early (15 weeks) and late (35 weeks) models of CRC induced by AOM in rats. The results were also correlated with the types and sizes of lesions. A better understanding of the roles of activins and their related molecules in colonic tumorigenesis may result in the development of more effective early diagnostic and/or therapeutic modalities for this common and deadly malignancy.

## Methods

### Study design

The study was approved by the Committee for the Care and Use of Laboratory Animals at Umm Al-Qura University. A total of 80 adult male Wistar rats of 10 weeks of age and 200–250 g/each were housed in clean and sterile polyvinyl cages (five rats/cage), maintained on standard laboratory pellet diet and water *ad libitum*; and kept in a temperature-controlled air-conditioned at 22–24 °C and 12 h dark/light cycle. The rats were randomly categorised following acclimation for 1 week into 20 rats that served as ‘Control group’ and the remaining 60 animals were allocated equally for the 15 weeks ‘S-AOM’ group and 35 weeks ‘L-AOM’ group for the short and long studies, respectively. AOM (Sigma-Aldrich, MO, USA) was dissolved in normal sterile saline and injected subcutaneously into the animals at a dose of 15 mg/kg/week for a total of 2 weeks to induce colon neoplasia as previously described [23].

Euthanasia was carried out using diethyl ether (Fisher Scientific UK Ltd, Loughborough, UK) for anaesthesia and 3 ml of blood were immediately collected from each rat in a plain tube through the vena cava and the obtained serum were stored in −20 °C till used. The colon from each animal was resected, incised through its longitudinal axis and was then submerged in 10 % formalin overnight between layers of filter papers with the mucosa facing upwards. The surface area for each colon was calculated as follow: Length X Width in cm^2^. All specimens were then processed for gross and histopathological examinations and later for immunohistochemistry, ELISA and gene expression studies.

### Gross and microscopic quantification of tumours

The average numbers of tumours on the mucosal surface of each colon were calculated by naked eye examination by two observers and who were blind to the source of tissues. Each colon was then cut equally into proximal, middle and distal segments. All segments were stained with 0.2 % methylene blue solution for 1.5–2 min, examined by a dissecting microscope (Human Diagnostics, Germany) at × 20 magnification to calculate the numbers of small tumours that were not detected by gross examination as well as aberrant crypt foci (ACF) and flat ACF by 2 blinded examiners to the source animals and according to the previously published criteria [24]. The final numbers of micro-tumours and ACF were calculated by averaging the results of both observers. The surface areas of ACF and flat ACF were calculated in mm^2^ using ImageJ software (https://imagej.nih.gov/ij/) (Additional file 1: Figure S1) [25, 26].

Two colonic specimens of 15 mm length and 4 mm width from each of the 3 colonic segments (proximal, middle and distal)/rat were excised under the dissecting microscopy and the collected tissues were processed for histopathological and immunohistochemical experiments. One specimen was placed in cross-sectional orientation and the other for topographic view. The remaining tissues were kept in in 15 ml of RNA*Later* (Thermo Fisher Scientific, CA, USA) following distaining and preserved in −80 °C till processed for quantitative RT-PCR or total protein extraction by RIPA lysis buffer.

### Histopathological staining and examination

Tissue specimens from each colonic segment were processed by a conventional method, cut in 5 μm serial sections following embedding in paraffin, and stained by haematoxylin and eosin for histopathology. All sections were also stained according to the previously established protocol with 1 % Alcian blue (AB) in 3 % acetic acid followed by Neutral red counterstaining for the detection of mucin depleted foci (MDF) [27, 28].

The glandular cellular morphology as well as the numbers of ACF/MDF were examined on an EVOS XL Core microscopy (Thermo Fisher Scientific). MDF were characterised by the absence of blue staining within colonic goblet cells of aberrant crypt foci as previously described [27, 28]. ACF were microscopically classified according to the previously established criteria into hyperplastic or dysplastic [23]. Colonic adenomas consisted of proliferative/hyperplastic colonic glands, while adenocarcinoma was characterised by dysplastic glands that invaded the submucosal muscle layer [22]. All the lesions were characterised and counted in five random fields from each section by an expert histopathologist who was blind to the specimen group. The total numbers of ACF and MDF per colon were calculated by summing the results from the 3 segments of each colon. The surface areas of MDF (×200 magnification), adenoma and adenocarcinomas (×100 magnification) were calculated in μm^2^ (Additional file 2: Figure S2) using ImageJ [25, 26].

### Immunohistochemistry

Primary polyclonal rabbit IgG antibodies (Santa-Cruz Biotechnology Inc., CA, USA) were used for the detection of activin βA-subunit, βB-subunit, ACTRIIA, ACTRIIB, phosphorylated (p)-smads 2&3, smad4, smads 6&7 and follistatin. Noteworthy, the antibody against smad6 &7 does not differentiate between both types. An avidin-biotin horseradish peroxidase technique was applied to localise the molecules of interest using ImmunoCruz™ Rabbit LSAB Staining System (Santa-Cruz Biotechnology Inc.) and by following the manufacturer’s protocol. The concentration was 1:100 for both activin type II receptors, follistatin and smad4 antibodies while a concentration of 1:50 was used for the remaining antibodies. The negative control slides consisted of a section of the tissue block being studied, which was treated identically to all other slides, with the exception that the primary antibodies were omitted to control for non-specific binding of the detection system.

The sections were observed on an EVOS XL Core microscope at × 100, ×200 and × 400 magnifications to evaluate and score the immunostain. Each section was examined by two observers who were blind to the source of tissue and the intensity of staining was assessed in 5 random fields of each section at × 200 magnification and by using ‘H score’ that was calculated as follow [23, 29]: H score = ƩP_ί_ (ί +1), where ί represents the intensity of staining (0 = negative; 1 = weak; 2 = moderate and 3 = strong) and P_ί_ is the percentage of cells (0–100 %) stained at each intensity. In the case of a wide disagreement between both observers, the slides were reanalysed by a third independent reviewer.

### Quantitative RT-PCR

The cDNA was synthesised by transcribing 200 ng of total RNA using a high capacity RNA-to-cDNA Reverse Transcription Kit (Thermo Fisher Scientific) according the manufacturer’s protocol. PCR reactions were carried out in triplicate wells on ABI® 7500 system using power SYBR Green master mix (Thermo Fisher Scientific). The PCR reaction for each well included 10 μl SYBR Green, 7 μl DNase/RNase free water, 1 μl of each primer (5 pmol) and 1 μl cDNA (25 ng) and, 40 cycles (95 °C/15 s and 60 °C/1 min) of amplification were performed. Two negative controls were included, one with minus-reverse transcription (minus-RT) control from the previous RT step and a minus-template PCR, in which nuclease free water was used as a template.

The 2^-∆∆Ct^ method was used to perform relative quantitative gene expression of rat *INHBA*, *INHBB*, *ACVR2A*, *ACVR2B*, *FST*, *Smad2*, *Smad3*, *Smad4*, *Smad6* and *Smad7* target genes. Three reference genes were tested and rat *β-actin* gene showed the most consistent results and it was used to normalise the Ct values of the genes of interest. The results are expressed as fold-change compared with the control group. All used primers (Additional file 3: Table S1) were designed in-house and previously validated [29].

### Enzyme linked immunosorbant assay (ELISA)

Two colonic tissue specimens of 50 mg each that involved tumours (except for the control group) were submerged in 2 ml RIPA lysis buffer with protease inhibitors (Santa-Cruz Biotechnology Inc.) for protein extraction using electrical homogeniser. All homogenated samples were centrifuged at 14,000 rpm for 30 min at 4 °C and small aliquots (0.5 ml) of the resultant supernatant were placed in Eppendorf tubes. The concentrations of total proteins in the colonic tissue homogenates were measured at 280 OD on a BioSpec-nano machine (Shimadzu Corporation, Japan). All protein samples were diluted by normal sterile saline for a final concentration of 500 μg/ml of total protein.

The concentrations of activins and follistatin in serum and tissue homogenates were measured using specific ELISA kits (Cloud-Clone Corp., Houston, USA). All samples were processed in duplicate on a fully automated system (Human Diagnostics, Germany) and by following the manufacturer’s instructions. The detection ranges were between 12.3 and 1000 pg/mL for both activin-A & -B, 15.62–1000 pg/mL for activin-AB and 3.12–200 ng/mL for follistatin. The minimal detectable concentrations were 4.66 pg/mL for activin-A, 4.64 pg/mL for activin-B, 5.6 pg/mL for activin-AB and 1.23 ng/mL for Follistatin. All kits had intra-assay and inter-assay precisions of <10 % and <12 %, respectively.

### Statistical analysis

SPSS version 16 was used for the statistical analysis of the results and, normality and homogeneity of data were assessed by the Kolmogorov-Smirnov test and Levene test, respectively. Student’s *t* test or Mann-Whitney *U* test was used to compare between 2 groups based on data normality. One way ANOVA followed by LSD post hoc test were used to compare between the 3 groups. Correlations were determined by Pearson’s test. *P* value < 0.05 was considered significant.

## Results

### Gross and histopathological features of AOM induced colonic lesions

**Fig. 1:**
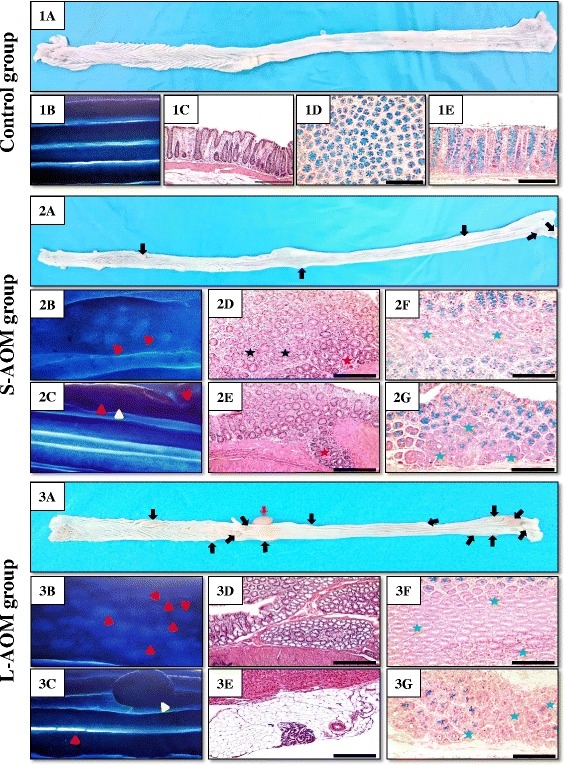
Features of colon mucosa by naked eye examination in (1A) control, (2A) S-AOM and (3A) L-AOM groups. Stained colonic mucosa with 0.2 % methylene blue from (1B) control, (2B & 2C) S-AOM and (3B & 3C) L-AOM groups to identify ACF and micro-tumours on the mucosal surface by dissecting microscope (×20 magnification). Colonic tissue sections from (1C) control, (2D & 2E) short and (3D & 3E) long groups to characterise the microscopic features at × 100 magnification following H&E stain. MDF were characterised at × 200 magnification and following staining with 1 % Alcian blue and Neutral red stains in (1D & 1E) normal, (2F & 2G) S-AOM and (3F & 3G) L-AOM in cross-sectional and topographic views, respectively. (*Black arrow* = tumour observed by naked eye; *red arrow* = regional lymph node enlargement; *red arrow head* = flat ACF; *yellow arrow head* = micro-tumour detected by dissecting microscope; *black star* = large ACF [>4 crypts/focus] with hyperplasia; *red star* = large ACF with dysplastic features and green star = MDF)

None of the rats from all groups died during the study and tumours were detected by gross examination on the mucosal surface of colons collected from both the short and long study groups (Fig. 1; panels 1A, 2A and 3A). The numbers of grown tumours detected by naked eye was significantly higher (*P* = 0.003) in the L-AOM compared with the S-AOM group (Table 1). Notably, enlargement of regional lymph nodes (Fig. 1; panel 3A) was also observed in 5 rats (16.7 %) of the L-AOM group.

**Table 1:** Mean ± SD of body weight, colon surface area (length X width in cm), count of colonic tumours by gross and dissecting microscope, number of tumour/colon surface area ratio (NT/CS), numbers and median surface areas of large (≥4 crypts/focus) and flat ACFs, adenoma and adenocarcinoma in the different study groups

			Control group (*n* = 20)	S-AOM group (*n* = 30)	L-AOM group (*n* = 30)
Body weight (g)			231.57 ± 20.01	221.97 ± 23.01	179.6 ± 15.64^b,d^
Colon surface area (cm^2^)			19.1 ± 2.2	18.89 ± 3.43	17.81 ± 1.84^a^
Tumour count		Gross	ND	7.2 ± 2.9	12.5 ± 3.21^d^
		Dissecting Microscope	ND	15.4 ± 5.1	24.8 ± 5.9^d^
		Total	N/A	23.1 ± 3.7	35.3 ± 4.1^d^
NT/CS Ratio			N/A	1.33 ± 0.4	1.95 ± 0.2^c^
Dissecting microscope (×20 magnification)	large ACF	Numbers	1.1 ± 0.2	69.4 ± 10.2^b^	143.2 ± 26.1^b,d^
		Median surface area (mm^2^)	4.1 (range 2.4–7.1)	29.7 (range 7.3–42.4)^b^	85.3 (range 7.9–133.3)^b,d^
	Flat ACF	Numbers	ND	18.4 ± 7.3	31.2 ± 9.6^c^
		Median surface area (mm^2^)	N/A	21.2 (range 8–51.7)^b^	69.4 (range 9.3–91.8) ^d^
Light microscope	MDF (×200 magnification)	Numbers	ND	17.4 ± 3.1	29.1 ± 3.6^d^
		Median surface area (μm^2^)	N/A	161.5 (range 12.2–352.9)	596.4 (range 142.8–966.3)^d^
	Adenoma (×100 magnification)	Numbers	ND	9.8 ± 3.1	11.6 ± 2.8
		Median surface area (μm^2^)	N/A	353.6 (range 101.7–507.8)	728.3 (range 274.8–1396.7)^d^
	Adenocarcinoma (×100 magnification)	Numbers	ND	16.7 ± 4.1	82.6 ± 15.7^d^
		Median Surface area (μm^2^)	N/A	107.9 (range 48–251.7)	711.8 (range 222.8–1808.4)^d^

*N/A* not applicable, *ND* not detected, ^a^ *P* < 0.05 compared with control; ^b^ *P* < 0.01 compared with control, ^c^ *P* < 0.05 compared with S-AOM group and ^d^ *P* < 0.01 compared with S-AOM group

Examination under dissecting microscope following methylene blue staining showed normal mucosal and crypt appearance in the control group (Fig. 1; panel 1B). A significant increase in the numbers of large ACF (>4 crypts/focus) in both the short (*P* = 0.0001) and long (*P* = 0.03 × 10^−5^) compared with controls was also observed (Table 1). Flat ACF was also detected in both AOM groups, but not the control, and the numbers were significantly higher (*P* = 0.002) in long (Fig. 1; Panel 3B) compared with short (Fig. 1; Panel 2B) AOM groups (Table 1). Similarly, the numbers of micro-tumours detected by dissecting microscope (Table 1) were significantly higher (*P* = 0.006) and had larger surface areas (*P* = 0.07 × 10^−4^) in the L-AOM (Fig. 1; Panel 3C) compared with S-AOM group (Fig. 1; Panel 2C).

MDF were present in the 2 groups of AOM either in topographic (Fig. 1; panels 2F and 3F) or cross-sectional (Fig. 1; panels 2G and 3G) orientations. The numbers (*P* = 0. 02) and sizes (*P* = 0.0004) of MDF were significantly higher in the L-AOM compared with S-AOM group (Table 1). Additionally, large MDF (>12 aberrant crypts/focus) were detected in both groups of AOM and greater numbers of crypts/focus were associated with the L-AOM group, reaching to more than 50+ crypts/focus (Fig. 1; panel 3F) compared with 20+ in the S-AOM group (Fig. 1; panel 2F). Interestingly, cross-sectional specimens showed several large MDF that were located at the bottom end of the mucosa and beneath luminal mucin-secreting glands (Fig. 1; panel 2G). All detected MDF at the bottom end of colonic mucosa showed features of high grade dysplasia (Fig. 1; panels 2F, 2G and 3F). Additionally, the majority of adenocarcinomas in the L-AOM group were mucin depleted (Fig. 1; panel 3G).

Multiple tubular adenomas that consisted of several hyperplastic as well as dysplastic large ACF (>4 crypts/focus) were observed by histopathology in the ‘S-AOM’ group (Fig. 1; panels 2 D & E). Growing in situ carcinoma were also detected in several rats (*n* = 7) within the group and were characterised by large high-grade dysplastic ACF invading the lamina propria but not reaching the muscularis mucosae (Fig. 1; panel 2E). Oppositely, multiple adenocarcinomas/animal were seen in the L-AOM group (Fig. 1; panel 3D). Moreover, metastasis in the colonic serosa was seen in 6 rats (20 %) of the group. The colonic serosa was identified microscopically by the mesothelial cells and the detected metastatic foci were characterised by the presence of well to moderately differentiated colonic glands within the fatty subserosal stroma located beneath the mesothelium (Fig. 1; panel 3E).

### Immunohistochemical characteristics of all molecules in pre-neoplastic, benign and malignant colonic lesions

**Fig. 2:**
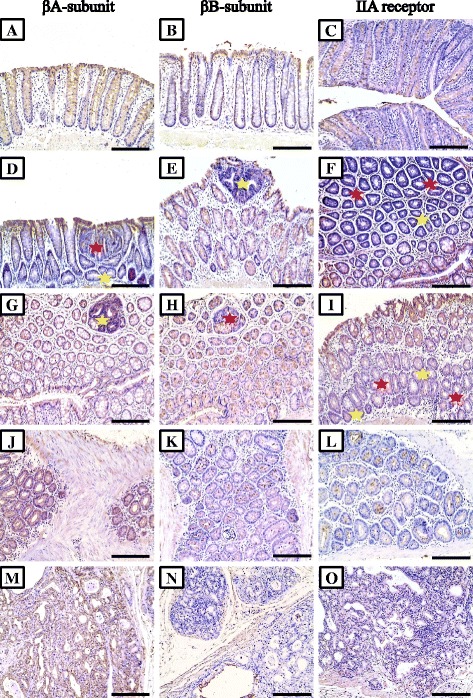
Immunohistochemical expression of activin βA-subunit (*left column*), βB-subunit (*middle column*) and type IIA receptor (*right column*) in normal colonic mucosa from control (**a**, **b** & **c**), pre-neoplastic lesions from S-AOM group (**d** to **i**), adenocarcinoma (**j**, **k** & **l**) and serosal metastatic foci (**m**, **n** & **o**) from the L-AOM group. (*Red star* = large dysplastic ACF and *yellow star* = MDF; ×200 magnification, scale bar = 8 μm)

**Fig. 3:**
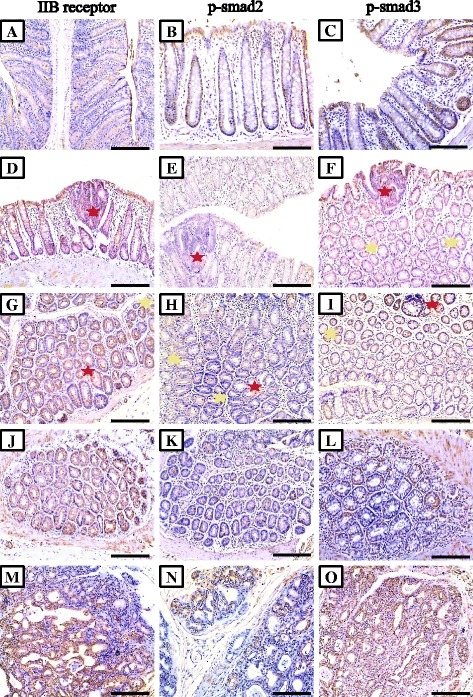
Immunohistochemical expression of activin IIB receptor (*left column*), phosphorylated smad2 (*middle column*) and smad3 (*right column*) in normal colonic mucosa from control (**a**, **b** & **c**), pre-neoplastic lesions from S-AOM group (**d** to **i**), adenocarcinoma (**j**, **k** & **l**) and serosal metastatic foci (**m**, **n** & **o**) from the L-AOM group. (*Red star* = large dysplastic ACF and *yellow star* = MDF; ×200 magnification, scale bar = 8 μm)

**Fig. 4:**
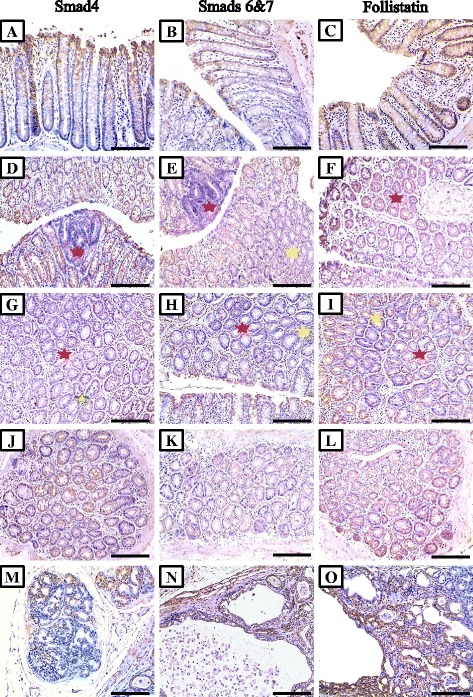
Immunohistochemical expression of smad4 (*left column*), smads 6&7 (*middle column*) and follistatin (*right column*) in normal colonic mucosa from control (**a**, **b** & **c**), pre-neoplastic lesions from S-AOM group (**d** to **i**), adenocarcinoma (**j**, **k** &** l**) and serosal metastatic foci (**m**, **n** & **o**) from the L-AOM group. (*Red star* = large dysplastic ACF and *yellow star* = MDF; ×200 magnification, scale bar = 8 μm)

All target molecules were detected in normal colonic glandular epithelium and showed cytoplasmic localisation, except for p-smad2 (Fig. 3; middle column), p-smad3 (Fig. 3; right column) and smad4 (Fig. 4; left column) which also showed nuclear staining. Alterations in the immunostain characteristics of the molecules of interest were seen in the 2 AOM groups and were lesion-dependent (Table 2). Overall, Significantly higher expression of activin βA-subunit (Fig. 2; left column) were seen in the S-AOM (257.1 ± 33.3; *P* = 0.03) and L-AOM (322.2 ± 37.7; *P* = 0.006) compared with control group (215.5 ± 31.1). Additionally, there was a significant difference (*P* = 0.007) between the early (Fig. 2; panels d a and g) and late (Fig. 2; panels j and m) models of CRC in the expression of βA-subunit. Similarly, activin type IIB receptor (Fig. 3; left column) and follistatin (Fig. 4; left column) were significantly increased in the S-AOM (264.4 ± 31.3; *P* = 0.007 and 318.8 ± 38.7; *P* = 0.002, respectively) and L-AOM (357.2 ± 36.4; *P* = 0.008 and 347.7 ± 32.8; *P* = 0.005, respectively) groups compared with controls (116.6 ± 26.7 and 237.6 ± 32.2, respectively). Significant alterations were also detected for ACTRIIB (*P* = 0.03) and follistatin (*P* = 0.01) between both AOM groups.

**Table 2:** Mean ± SD of immunohistochemistry scores for activin βA- and βB-subunits, type 2 receptors, phosphorylated (p)-smads2&3, Smad4, smads6&7 and follistatin proteins in colon specimens from the different groups and according to pre-neoplastic (large ACF and MDF), benign and malignant lesions

	Control group	S-AOM group			L-AOM group	
	*Normal gland*	*Pre-neoplastic lesion*	*Adenoma*	*Carcinoma* in situ	*Carcinoma*	*Serosal metastatic carcinoma*
βA-subunit	215.5 ± 31.1	296.8 ± 34.4^b^	255.1 ± 29.3^a,c^	313.3 ± 22.2^b,d^	334.1 ± 27.2^b,c,d,e^	363.7 ± 34.3^b,c,d,e,f^
βB-subunit	173.7 ± 28.7	113.4 ± 29.2^b^	227.2 ± 33.2^a,c^	129 ± 31.4^b,d^	122.8 ± 25.5^b,c,d^	73.7 ± 21 .9^b,c,d,e,f^
ACTRIIA	201.1 ± 26	137.2 ± 28.3^b^	234.7 ± 31.7^a,c^	103.3 ± 28.3^b,c,d^	93.4 ± 22.2^b,c,d^	68.8 ± 23.3^b,c,d,e,f^
ACTRIIB	116.6 ± 26.7	261.8 ± 37.7^b^	277.6 ± 31.6^b^	311.4 ± 22.8^b,c,d^	321.3 ± 28.9^b,c.d^	352.4 ± 27.6^b,c,d,e,f^
p-Smad2	192.4 ± 29.5	137.1 ± 26.8^b^	88.8 ± 21.7^b,c^	79.6 ± 25.3^b.c^	78.5 ± 22.9^b,c^	81.3 ± 18.8^b,c^
p-Smad3	272.7 ± 27.6	236.5 ± 42.6^a^	228.4 ± 47.4^b^	142.8 ± 43.6^b,c,d^	133.3 ± 39.3^b,c,d^	314.7 ± 32.2^a,c,d,e,f^
Smad4	226.7 ± 29.4	163.1 ± 27.2^a^	108.8 ± 33.1^b,c^	134.5 ± 35.3^b,c^	121.4 ± 38.4^b,c^	81.3 ± 22.2^b,c,d,e,f^
Samds6&7	166.8 ± 32.9	92.3 ± 21.7^b^	132.7 ± 31.3^a,c^	115.1 ± 21.3^a,d^	89.6 ± 23.3^b.d.e^	235.6 ± 42.2^b,c,d,e,f^
Follistatin	237.6 ± 22.2	272.3 ± 25.3^a^	291.4 ± 24.2^b^	317.8 ± 22.3^b,c,d^	333.8 ± 35.3^b,c,d^	365.8 ± 29.9^b,c,d,e^

^a^ *P* < 0.05 compared with normal; ^b^ *P* < 0.01 compared with normal; ^c^ *P* < 0.05 with pre-neoplastic lesion subgroup; ^d^ *P* < 0.05 compared with adenoma subgroup; ^e^ *P* < 0.05 with carcinoma in situ subgroup and ^f^ *P* < 0.05 compared with carcinoma subgroup

In contrast, a significant decrease was observed in the expression of activin βB-subunit (Fig. 2; middle column), activin type IIA receptor (Fig. 2; right column), p-smad2 (Fig. 3; middle column), p-smad3 (Fig. 3; right column), smad4 (Fig. 4; left column) and smads 6&7 (Fig. 4; middle column) in both short and long AOM compared with normal colons. Significantly lower expressions in the L-AOM in comparison with S-AOM samples were also observed for βB-subunit (93.9 ± 26.7 vs. 167.6 ± 51.1; *P* = 0.02), ACTRIIA (82.3 ± 17.9 vs. 189.6 ± 57.7; *P* = 0.03) and smad4 (111.4 ± 25.5 vs. 133.5 ± 32.1; *P* = 0.03). However, no difference (*P* > 0.05) was detected in p-smads 2&3 and smads 6&7.

By further analysis according to the type of colonic lesions, there was a significant consistent increase in the expression of activin βA-subunit, type IIB receptor and follistatin between pre-neoplastic, benign and malignant lesions. The expression values of all molecules according to lesion types are summarised in Table 2.

### Quantitative gene expression of activins and their related molecules

**Fig. 5:**
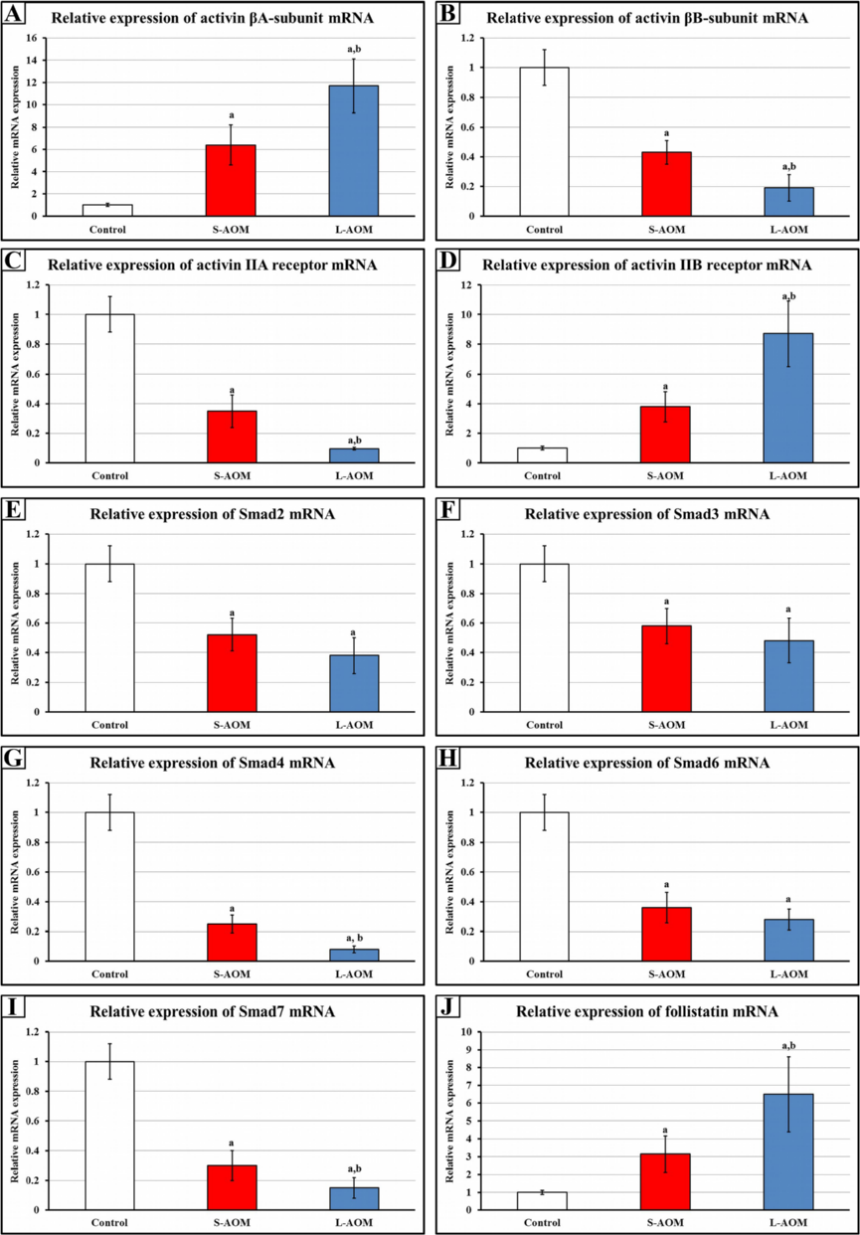
Mean ± SD of messenger RNA relative expression of **a** βA-subunit, **b** βB-subunit, **c** IIA receptor, **d** IIB receptor, **e** smad2, **f** smad3, **g** smad4, **h** smad6, **i** smad7 and **j** follistatin the different study groups. (a = *P* < 0.05 compared with control group; b = *P* < 0.05 compared with S-AOM group)

Gene expression studies showed a significant increase in the mRNA expression of *INHBA* (6 folds; *P* = 0.001), *ACVR2B* (10.8 folds; *P* = 0.0003) and *FST* (3.1 folds; *P* = 0.02), and a significant decrease in the gene expression of *INHBB* (2.3 folds; *P* = 0.02), *ACVR2A* (3 folds; *P* = 0.003), *smad2* (2.5 folds; *P* = 0.007), *smad3* (2 folds; *P* = 0.03), *smad4* (4 folds; *P* = 0.0004), *smad6* (3 folds; *P* = 0.003) and *smad7* (3.2 folds; *P* = 0.005) in the S-AOM group when compared with the control tissues. The gene expression of all candidate molecules, except for smads 2, 3 and 6, were also significantly altered in the L-AOM compared with the S-AOM group (Fig. 5).

### Tissue and serum concentrations of mature activin proteins and follistatin

The tissue homogenate concentrations of mature activin-A, activin-AB and follistatin, but not activin-B, proteins were significantly different between the 3 study groups. A significant increase in activin-A (*P* = 0.004) and follistatin (*P* = 0.06 × 10^−3^), while a significant decrease in activin-AB (*P* = 0.03) were detected in the colon tissue homogenates from the S-AOM compared with control group. A further increase in both activin-A (*P* = 0.0003) and follistatin (*P* = 0.02) as well as a further decrease in activin-AB (*P* = 0.03) was also statistically significant in the tissue homogenates of L-AOM compared with S-AOM group.

At the serum level, significant alterations were only detected in the concentrations of activin-A (*P* = 0.007) and follistatin (*P* = 0.03) between the short and control groups. Serum samples from the L-AOM group had also significantly higher concentrations of activin-A (*P* = 0.01) and follistatin (*P* = 0.02) compared with the S-AOM group. There was no significant change (*P* > 0.05) by one way ANOVA in the serum concentrations of both activin-B and activin-AB between the study groups (Table 3).

**Table 3:** Mean ± SD of protein concentrations of activin-A, activin-B, activin-AB and follistatin in serum and colon tissue homogenates by ELISA

		Normal group	S-AOM group	L-AOM group
Activin-A (pg/mL)	*Tissue*	388.8 ± 61.7	586.4 ± 72.6^b^	862.3 ± 210.2^b,d^
	*Serum*	266.5 ± 41.7	472.4 ± 63.3^b^	614.8 ± 111.4^b,c^
Activin-B (pg/mL)	*Tissue*	337.8 ± 68	298.1 ± 54.7	315.2 ± 47.3
	*Serum*	180.4 ± 43.3	191.2 ± 32.8	213.9 ± 56.5
Activin-AB (pg/mL)	*Tissue*	277.4 ± 34.2	154.7 ± 41.4^a^	83.4 ± 24.2^b,c^
	*Serum*	181.7 ± 62.8	172.9 ± 40.9	202.2 ± 77.8
Follistatin (ng/mL)	*Tissue*	5.6 ± 1.8	15.2 ± 3.9^b^	38.01 ± 6.2^b,d^
	*Serum*	3.83 ± 1.1	7.4 ± 2.3^b^	11.2 ± 2.9^b,c^

(^a^ *P* < 0.05 compared with control; ^b^ *P* < 0.01 compared with control, ^c^ *P* < 0.05 compared with S-AOM group and ^d^ *P* < 0.01 compared with S-AOM group)

Correlation studies showed significant strong positive correlations for tissue activin-A and follistatin, and a significant inverse correlation for tissue activin-AB with the numbers and surface areas of large and flat ACF, MDF, adenocarcinoma as well as the numbers of gross and micro-tumours (Table 4).

**Table 4:** Results of correlation analysis using Pearson’s test for tissue and serum concentrations of activin-A, activin-B, activin-AB and follistatin with the numbers (N) and surface areas (SA) of the different types of colonic lesions (*P < 0.05)

			Large ACF		Flat ACF		MDF		Adenoma		Carcinoma		Tumours	
			N	SA	N	SA	N	SA	N	SA	N	SA	Gross	Micro
Activin-A (pg/mL)	Tissue	*R Value*	0.123	0.271^*^	0.364^*^	0.288^*^	0.416^*^	0.383^*^	0.027	0.091	0.589^*^	0.471^*^	0.389^*^	0.352^*^
		*P Value*	0.7	0.008	0.03	0.004	0.0001	0.003	0.9	0.8	0.003	0.02	0.02	0.03
	Serum	*R Value*	0.082	0.071	0.101	0.089	0.091	0.062	0.051	0.009	0.397^*^	0.312^*^	0.256^*^	0.299^*^
		*P Value*	0.8	0.8	0.7	0.8	0.7	0.9	0.9	1	0.03	0.04	0.03	0.03
Activin-B (pg/mL)	Tissue	*R Value*	−0.091	−0.103	−0.053	−0.071	−0.304^*^	−0.218^*^	−0.086	−0.091	−0.205^*^	−0.171	−0.005	−0.091
		*P Value*	0.7	0.7	0.5	0.6	0.002	0.03	0.3	0.4	0.03	0.1	0.9	0.4
	Serum	*R Value*	0.008	0.062	−0.003	−0.098	−0.004	−0.062	0.054	0.003	−0.037	0.011	0.063	0.019
		*P Value*	0.9	0.8	0.8	0.8	0.9	0.7	0.7	0.9	0.5	0.7	0.6	0.5
Activin-AB (pg/mL)	Tissue	*R Value*	0.019	−0.032	0.062	0.051	−0.342^*^	−0.288^*^	0.079	0.009	−0.377^*^	−0.311^*^	−0.289^*^	−0.300^*^
		*P Value*	0.8	0.8	0.4	0.4	0.002	0.01	0.3	0.9	0.009	0.02	0.007	0.006
	Serum	*R Value*	0.002	0.007	0.011	−0.092	−0.003	0.044	−0.007	−0.009	−0.104	−0.012	−0.100	−0.112
		*P Value*	0.9	0.8	0.8	0.8	1	0.9	1	1	0.4	0.8	0.4	0.7
Follistatin (ng/mL)	Tissue	*R Value*	0.144	0.171	0.164^*^	0.118	0.389^*^	0.299^*^	0.007	0.009	0.498^*^	0.412^*^	0.311^*^	0.243^*^
		*P Value*	0.08	0. 4	0.03	0.4	0.001	0.005	0.8	0.8	0.002	0.004	0.02	0.03
	Serum	*R Value*	0.002	0.051	0.091	0.009	0.001	0.055	0.062	0.019	0.333^*^	0.302^*^	0.187^*^	0.111
		*P Value*	1	0.8	0.7	0.9	1	0.9	0.9	0.9	0.004	0.006	0.02	0.1

## Discussion

The present study simultaneously measured the expression of activins and their related proteins in rat colonic tissues collected from AOM-induced colon cancer and the results were compared with normal tissue obtained from controls. The results of the molecules of interest were further analysed between early and late stages of CRC and were also correlated with the different types and sizes of colonic neoplastic lesions.

AOM-induced CRC in murine is a well-recognised and frequently used experimental model that shares many of the molecular tumorigenic pathways underlying the common sporadic form of human colon malignancy [8, 22]. Herein, we used a variety of previously well-established pre-neoplastic lesions to assess the initiation and progression of cancer [30–33]. Our findings are in parallel with the previously published characteristics of premalignant and cancerous colonic lesions associated with AOM model [30–33] Additionally, they support the earlier suggestion that these lesions are time-dependent since the numbers and sizes of adenocarcinoma were significantly higher in the L-AOM group and a minority of animals had metastatic foci within the colonic serosa and/or enlargement of regional lymph nodes [34].

However, MDF were detected in the present study by a modified protocol using 1 % AB pH 2.5 in sectioned rather than non-sectioned colonic specimens [28]. Interestingly, AB staining of cross-sectional specimens showed several MDF that were localised beneath luminal colonic glands, which had normal morphology and mucin contents, suggesting that substantial numbers of these pre-neoplastic lesions could have been missed if examination was performed in non-sectioned specimens. Furthermore, the majority of glands in these deeply-situated MDF showed dysplastic features similar to those usually reported in MDF detected in un-sectioned specimens [32, 33]. We therefore propose that the loss of mucin secretion is initiated a the lower extremity of a hyperplastic mucosal layer and later spreads to involve luminal glands, which is aligned with the “shift upwards theory” for colon carcinogenesis [35]. However, more studies using Periodic Acid-Schiff with Alcian blue staining protocol for the detection and differentiation between neutral and acid mucins are required to support our hypothesis [36].

The available reports on the expression of activins and their related molecules in the intestine, especially colon, are few and the majority only focused on activin-A. In vitro studies have shown that activin βA-subunit is expressed in epithelial cells from human embryonic and rat small intestinal cells [37, 38]. Exogenous activin-A inhibited cell proliferation and induced differentiation of rat IEC-6 cells [37], decreased the growth of mice m-ICc12 cells [39] while stimulated the proliferation of colonic epithelial cells collected from developing rats [40]. Nevertheless, others failed to detect activin βA-subunit and/or showed weak immunostain in normal human colonic tissues despite the localisation of activin receptors within the same samples [19, 41].

However, a significant increase in the expression of βA-subunit has been shown in enterocytes from patients with inflammatory bowel disease [41]. Similarly, studies in mice also reported weak or no expression of βA-subunit in normal colonic glands and the induction of colitis resulted in a significant increase of the molecule [39, 42]. The expression of both type IIA and IIB receptors as well as smads 2&3 has also been detected in mice normal colon epithelial cells and, a significant increase in their production was noted during colitis and they were co-localised with activin subunits within the same cells [39]. Injecting follistatin in vivo also inhibited the progress of inflammation [39, 42], while overexpression of activin-A in vivo following injection of a plasmid DNA containing βA-subunit cDNA in mice resulted in sever intestinal inflammation [43]. Notably, weak intraepithelial localisation of βB-subunit in normal colon has been reported by a single study and the expression increased during colitis and was co-localised with the βA-subunit [39].

Our study is in agreement and correlates with the aforementioned reports since it demonstrated the expression of activin subunits, activin type II receptors, smads and follistatin at the gene and protein levels by normal rat colonic enterocytes. The co-localisation of both activins subunits with their receptors observed by Zhang et al. [39] and ours advocates that the colonic epithelial cells are cable of synthesising as well as controlling the biological activities of activin proteins and provide further support to the notion that activins are involved in the regulation of colonic cellular physiology in a paracrine/autocrine mode of action [37–43]. Furthermore, this study is the first to detect the three mature activin isoforms in tissue homogenates of rat normal colon. We therefore hypothesise that each of the mature activin proteins could have unique physiological function(s) in the regulation of colonic homeostasis since the results from gene knockout experiments have shown that activin subunits do not functionally intersect in all settings in vivo [44, 45]. Further studies are, however, still needed to explore and compare the effect(s) of the different mature activin isoforms on the biology of normal colonic epithelial cells.

Colon homeostasis involves the regeneration and maintenance of mucosal integrity and, both processes require continuous cell production from the stem cell niche located at the base of colonic crypts that later migrate upwards and differentiate to fully functioning cells [46]. Additionally, a delicate balance between cell production and differentiation as well as apoptosis is tightly regulated in the colon by several molecular pathways [47]. Dysregulation in these pathways following a variety of insults is believed to result in the development of colon malignancy [23, 46, 47].

In deed deregulation of colonic endogenous activin system could well be involved in colon carcinogenesis. Activins play crucial roles in many cellular homeostatic functions including cell proliferation and differentiation, wound repair and regulation of immune responses [10]. Pathological increase in activin βA-subunit protein and mRNA has been observed in malignant tissues obtained from patients with CRC and the expression was stage-dependent [19–21]. Furthermore, there was a significant positive correlation between the expression levels of *INHBA* mRNA and serum activin-A with lymph node metastasis and the progression of malignancy, respectively [20, 21]. The researchers have therefore proposed serum activin-A as a potential sensitive and specific prognostic marker for colon cancer [19–21]. Frameshift mutations in the *ACVR2* gene have also been associated with microsatellite instable colon neoplasms [16–18]. Mutation inactivations of all the smads involved in the intracellular canonical pathway shared by both activins and TGF-β have also been documented during the progression of CRC in human [48–50].

The current findings are in agreement with the previous studies since they showed a significant increase in the expression of βA-subunit as well as a significant decrease in type IIA receptor and all tested smads at the protein and gene levels in tissues obtained from rat colon cancer. Additionally, the highest expression of βA-subunit and, the lowest expression of IIA receptor, smad4 and smad7, was observed in the L-AOM group especially in metastatic foci. Moreover, significantly greater levels of ACTRIIB and follistatin concurred with the observed increase of βA-subunit in malignant tissues. Contrariwise, a significant downregulation of activin βB-subunit corresponded with the decrease of IIA receptor and the different smad proteins in the two AOM groups. At the level of mature proteins, there was an increase in activin-A as well as follistatin and a decrease in activin-AB concentrations in colonic tissue homogenates and both activin isoforms significantly and paradoxically correlated with the numbers and sizes of both pre- and cancerous lesions. These observations suggest that, similar to human, the AOM-induced colon cancer in rat is associated with a disruption of rat colonic endogenous activin-A system, which appears to be a pro-tumorigenic pathway of CRC in both species. In addition, this study provides additional support to the potential clinical value of serum activins-A as a prognostic marker during colon malignancy [19–21].

Remarkably, our findings also propose that mature activin-AB may have anti-carcinogenic activities since there were significant inverse correlations between the progression of colon cancer with the tissue concentrations of the protein. In this regards, gene deletion of activin βA- and βB-subunits resulted in completely different phenotypes and the insertion of *INHBB* in *INHBA* ^−/−^ mice partially restored the lost functions [44, 45]. Additionally, there is a tremendous gap in our knowledge and understanding of the potential physiological and pathological roles of the other activin isoforms (B & AB) in the different cells and tissues since the majority of studies were mainly conducted on activin-A only [10]. Nevertheless, a recent study has shown that patients with breast cancer and were positive for epidermal growth factor receptor 2/neu, which is a marker of carcinogenesis, had significantly lower serum concentrations of activin-AB [51]. Therefore, the role of the activin-AB in tumour biology merits further research to explore its potential biological and anti-tumorigenic activities in the human colon.

Moreover, gene deletion studies have demonstrated that *ACVR2* ^−/−^ null mice exhibited an entirely different phenotype from those mice lacking type IIB receptor [52, 53]. Interestingly, both models of activin type II receptors deficient mice also differed from those lacking activin βA- or βB-subunits, suggesting that activin ligands may possibly interact and signal through other different receptors [44, 45]. Another study has also demonstrated that the overexpression of activin βA-subunit was associated with a decrease in the mRNA levels of ACTRIIA in the testes of inhibin deficient mice [54]. Additionally, a pathological increase in activin-A and its type IIB receptor has been shown to induce cancer cachexia and the use of a receptor decoy for blocking the ACTRIIB lead to a significant restoration in the muscle mass and increase in the survival rates of treated animals [55]. Hence, we postulate that each of the mature activin proteins may well have a preferential downstream intracellular pathway during colon tumorigenesis, where over production of activin-A could favour the activation of receptor IIB while activin-AB may propagate its signal via IIA receptor. Additionally, the intracellular propagation of activin signals through each of the activin type II receptors may possibly results in paradoxical non-overlapping effects during the course of colon carcinogenesis since the activation of type IIA results in anti-tumorigenic activities while IIB receptor pathway is pro-carcinogenic [17, 18, 55].

The associations between elevated activin-A and the downregulation of smads appear to be complex, as one of them could be simultaneously a cause and the other a consequence. In this context, the findings of a recent study using a xenograft model of oesophageal cancer have shown that the pro- and anti-tumorigenic effects of activin-A are concentration-dependent [56]. Hence, a sustained pathological up-regulation in colonic activin-A could favour the activation of other non-canonical intracellular mediators, such as ERK, p38 and Akt, and thus leading to a downregulation in the none-utilised smads [57–59]. Alternatively, others have reported that malignant enterocytes develop resistance to the growth inhibitory effects of activin-A mainly by inducing mutations in the ACTRIIA or the smad4-dependent pathway [15, 16]. Additionally, restoration of activin IIA receptor in vitro resulted in smad4-dependent growth inhibitory effects and cell cycle arrest of cancerous enterocytes but also induced their migration following treatment with activin-A [17, 18]. Therefore, it could be postulated that inactivation of smads following gene mutations [48–50] may result in hyper-physiological concentrations of activin-A and subsequently the activation of non-canonical pathways that involve several pro-carcinogenic molecules [57–59]. Therefore, additional in vivo and in vitro studies are mandatory to explore the interactions between activins and their canonical and non-canonical signal mediators in normal and cancerous colonic cells.

Other proposed mechanisms for the development of resistance by tumour cells to the growth inhibitory effects of activin-A include up-regulation of follistatin [60, 61]. Our results showed significantly higher concentrations of follistatin at the gene and protein levels that positively correlated with the progression of colon cancer in the used model. Currently there is no report in the literature regarding the role of follistatin in colon cancer. Nevertheless, our results suggest that the observed increase in follistatin could be an independent pro-oncogenic molecule. In support of the previous suggestion, in vitro studies on prostate cancer have revealed that the progression of tumour and development of resistance to the growth inhibitory actions of activin-A were associated with higher levels of follistatin [62, 63]. Alternatively, the up-regulation of follistatin may plausibly be a compensatory mechanism against the pathological increase of activin-A since a more recent study has also reported that follistatin inhibited cancer progression in a subset of pancreatic cancer cell lines that are known to highly express both activin β-subunits [64].

## Conclusions

Normal rat colonic glandular epithelial cells are capable of synthesising and responding to the different mature activins isoforms in a paracrine/autocrine mode of action as well as tightly controlling the bioactivities of these ligands at both the extra- and intracellular levels. Additionally, colonic activin system is pathologically altered and could be involved in colon tumorigenesis and, the expression of activins appears to be time-dependent and lesion-specific. The significant correlations for serum and tissue activin-A and follistatin with the types and sizes of neoplastic lesions provide further support to the notion that these proteins could be sensitive biochemical markers and/or potential molecular targets for the diagnosis and/or treatment of colon cancer. Further studies are required to illustrate the clinical value of activins and their related proteins in human colon cancer.

## Additional files

Additional file 1: Figure S1. Steps of processing the study digital images with ImageJ software for calculating the surface areas of colonic micro-tumours (left column) and flat ACF (middle column) detected by dissecting microscopy following methylene blue staining; and MDF (right column) by light microscopy following staining with 1 % Alcian blue. The identification and selection of the areas of interest (2^nd^ row from top) were done with the guidance of an expert histopathologist. The images were then processed using hue/saturation/brightness (HSB) for colour threshold adjustment using ‘red’ as the threshold colour to digitally mark and select an area of interest by the software (3^rd^ row from top). This was followed by transforming all the images to binary colours to ensure that only the areas of interest were precisely defined and selected by the software (bottom row). All measurements were calculated following calibration with digital photos of corresponding microscopic scale slides captured at the designated magnifications. (Panels 1A-E and 2A-E: ×20 magnification, scale bar = 2 mm; panels 3A-E: ×200 magnification, scale bar = 8 μm). (PPTX 1499 kb)
Additional file 2: Figure S2. Steps of processing the study digital images with ImageJ software for calculating the surface areas of colonic adenoma (left column; ×100 magnification), carcinoma in situ (middle column; ×100 magnification) and adenocarcinoma (right column; ×200 magnification) observed by light microscopy and following staining with haematoxylin & eosin. The areas of interest were also selected with the support of an expert histopathologist (2^nd^ row from top), then processed for colour threshold adjustment using HSB and ‘red’ as threshold colour (3^rd^ row from top) and, finally all the images were transformed to binary colours in which the areas of interest appear in solid black colour (bottom row). All measurements were calculated following calibration with digital photos of corresponding microscopic scale slides captured at the designated magnifications. (Panels 1A-E and 2A-E: ×100 magnification, scale bar = 15 μm; panels 3A-E × 200 magnification, scale bar = 8 μm). (PPTX 1748 kb)
Additional file 3: Table S1. Contains the sequence of forward and reverse primers for the detection of target genes along with the amplicon sizes. (DOC 44 kb)
Additional file 4: Histopathology, immunohistochemistry, ELISA and gene expression raw data. Raw data generated from gross and microscopic studies of tumours, digital image analysis for measuring the surface areas of neoplastic lesions, immunohistochemistry scores of all studied molecules, ELISA data generated from serum and colonic tissues; and data form quantitative expression of all targeted genes. (XLSX 674 kb)

## Abbreviations

AB: Alcian blue
ACF: Aberrant crypt foci
ACTRIIA: Activin type IIA receptor
ACTRIIB: Activin type IIB receptor
Akt: Protein kinase B
AOM: Azoxymethane
CRC: Colorectal carcinoma
ERK: Extracellular signal-regulated kinases
MDF: Mucin depleted foci
p38: Mitogen-activated protein kinases
TGF-β: Transforming growth factor
