# Supplementary figures and images for: Activins and their related proteins in colon carcinogenesis: insights from early and advanced azoxymethane rat models of colon cancer
Source: BMC Cancer. 2016 Nov 11;16:879. doi: 10.1186/s12885-016-2914-9 (PMC5106801; doi:10.1186/s12885-016-2914-9)

## Slide 1
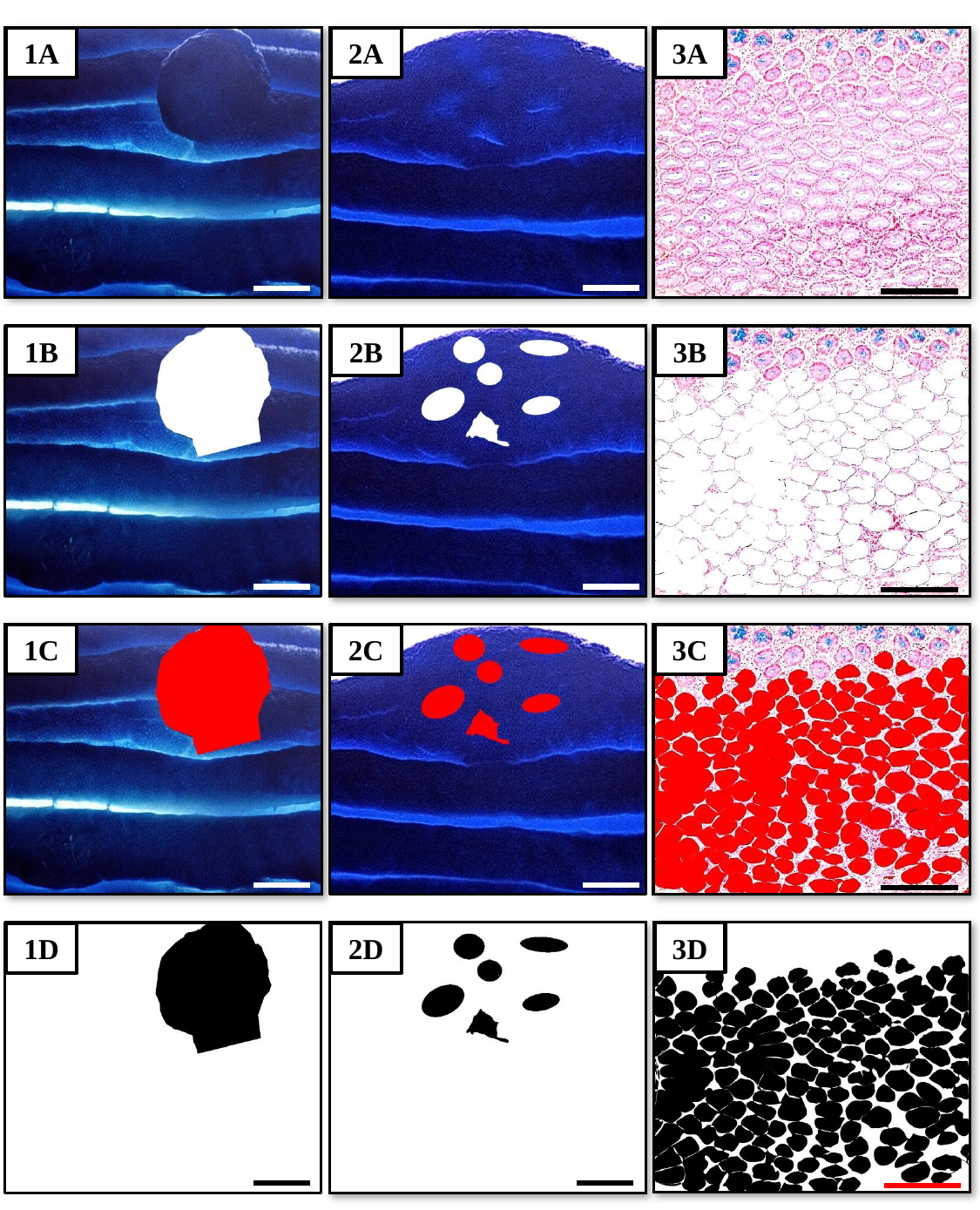

1A
2A
3A
1B
2B
3B
1C
2C
3C
3D
1D
2D

Supplement: Additional file 1: Figure S1. — Steps of processing the study digital images with ImageJ software for calculating the surface areas of colonic micro-tumours (left column) and flat ACF (middle column) detected by dissecting microscopy following methylene blue staining; and MDF (right column) by light microscopy following staining with 1 % Alcian blue. The identification and selection of the areas of interest (2nd row from top) were done with the guidance of an expert histopathologist. The images were then processed using hue/saturation/brightness (HSB) for colour threshold adjustment using ‘red’ as the threshold colour to digitally mark and select an area of interest by the software (3rd row from top). This was followed by transforming all the images to binary colours to ensure that only the areas of interest were precisely defined and selected by the software (bottom row). All measurements were calculated following calibration with digital photos of corresponding microscopic scale slides captured at the designated magnifications. (Panels 1A-E and 2A-E: ×20 magnification, scale bar = 2 mm; panels 3A-E: ×200 magnification, scale bar = 8 μm). (PPTX 1499 kb) [file 12885_2016_2914_MOESM1_ESM.pptx]

## Slide 1
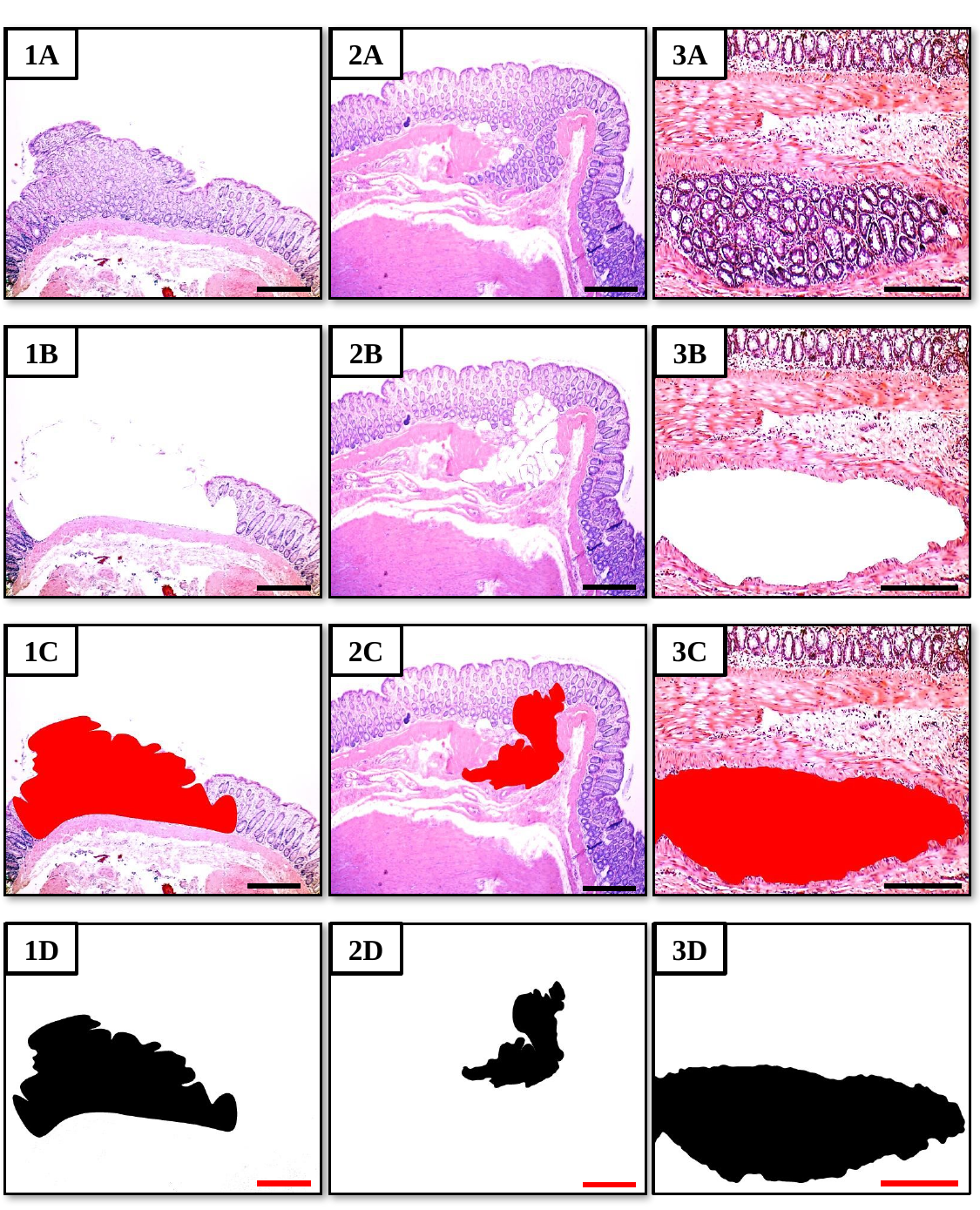

2A
3A
1A
1B
2B
3B
1C
2C
3C
1D
2D
3D

Supplement: Additional file 2: Figure S2. — Steps of processing the study digital images with ImageJ software for calculating the surface areas of colonic adenoma (left column; ×100 magnification), carcinoma in situ (middle column; ×100 magnification) and adenocarcinoma (right column; ×200 magnification) observed by light microscopy and following staining with haematoxylin & eosin. The areas of interest were also selected with the support of an expert histopathologist (2nd row from top), then processed for colour threshold adjustment using HSB and ‘red’ as threshold colour (3rd row from top) and, finally all the images were transformed to binary colours in which the areas of interest appear in solid black colour (bottom row). All measurements were calculated following calibration with digital photos of corresponding microscopic scale slides captured at the designated magnifications. (Panels 1A-E and 2A-E: ×100 magnification, scale bar = 15 μm; panels 3A-E × 200 magnification, scale bar = 8 μm). (PPTX 1748 kb) [file 12885_2016_2914_MOESM2_ESM.pptx]
