# Supplementary material for: Activins and their related proteins in colon carcinogenesis: insights from early and advanced azoxymethane rat models of colon cancer
Source: BMC Cancer. 2016 Nov 11;16:879. doi: 10.1186/s12885-016-2914-9 (PMC5106801; doi:10.1186/s12885-016-2914-9)
Supplement: Additional file 3: Table S1. — Contains the sequence of forward and reverse primers for the detection of target genes along with the amplicon sizes. (DOC 44 kb) [file 12885_2016_2914_MOESM3_ESM.doc]

**Supplementary table 1:** The sequences of PCR primers used for the detection of rat β-actin, β2 microglobulin, GAPDH, activin βA- and βB-subunits, activin type IIA and IIB receptors, smads 2/3/4/6/7 and follistatin mRNAs in colon samples including the corresponding genes accession numbers and amplicon sizes.

| **Genes** | **Forward** | **Reverse** | **Amplicon size** |
| --- | --- | --- | --- |
| ***ACTB***  (NCBI: NM_031144.3) | 5’ CGG TCA GGT CAT CAC TAT CG 3’ | 5’ TTC CAT ACC CAG GAA GGA AG 3’ | 79 bp |
| ***B2M***  (NCBI: NM_012512.2) | 5’ TGA AGG AGC CCA AAA CCG TC 3’ | 5’ CCG GAT CTG GAG TTA AAC TGG 3’ | 92 bp |
| ***GAPDH***  (NCBI: NM_017008.4) | 5’ GCA TCT TCT TGT GCA GTG CC 3’ | 5’ GAG AAG GCA GCC CTG GTA AC 3’ | 105 bp |
| ***INHBA***  (NCBI: NM_017128.2) | 5’ TGG GGG AAA ACG GGT ATG TG 3’ | 5’ CCT GAC TCG GCA AAG GTG AT 3’ | 106 bp |
| ***INHBB***  (NCBI: NM_080771.1) | 5’ GAG CGC GTC TCT GAG ATC AT 3’ | 5’ AGG TTC TGG TTG CCT TCG TT 3’ | 101 bp |
| ***ACVR2A***  (NCBI: NM_031571.2) | 5’ ATG GCC ACA AGC CTG CAA TA 3’ | 5’ AGA CTT GCC AGC CTC AAA CT 3’ | 122 bp |
| ***ACVR2B***  (NCBI: NM_031554.1) | 5’ TGG ACA TCC ATG AGG ACC CTG 3’ | 5’ ACC TCG AGC CTT GAT CTC CA 3’ | 89 bp |
| ***Smad2***  (NCBI: NM_019191.2) | 5’ GAA GGA ACA AAA GGT CCG GG 3’ | 5’ ATC CAC ACT CAG AAA CAG CCT 3’ | 92 bp |
| ***Smad3***  (NCBI: NM_013095.3) | 5’ CAT CAC CAC GCA GAA CGT GA 3’ | 5’ CCC TTC CGA TGG GAT ACC TG 3’ | 84 bp |
| ***Smad4***  (NCBI: NM_019275.3) | 5’ AAA GGT GAA GGG GAC GTG TG 3’ | 5’ CGG GTA GAT CTT GTG GAC GG 3’ | 120 bp |
| ***Smad6***  (NCBI: NM_001109002.2) | 5’ TCT ACG ACC TAC CTC AGG GC 3’ | 5’ CTG CGC TGC TCC AGG TT 3’ | 61 bp |
| ***Smad7***  (NCBI: NM_030858.1) | 5’ GGA GGT CAT GTT CGC TCC TT 3’ | 5’ TTT GGT CCT GAA CAT GCG GG 3’ | 74 bp |
| ***FST***  (NCBI: NM_012561.2) | 5’ TGT GAA GAC ATC CAG TGC GG 3’ | 5’ GAC GGG CTC ATC CGA CTT AC 3’ | 117 bp |
